# Detection of SARS-CoV-2 IgG antibodies in breast milk and serum of immunized lactating mothers

**DOI:** 10.1515/almed-2021-0077

**Published:** 2021-11-03

**Authors:** Sidra Sadiq, Faheem Arslan

**Affiliations:** Department of Clinical Chemistry & Endocrinology, Rehman Medical Institute, Peshawar, Khyber Pakhtunkhwa, Pakistan; Department of Radiology, Pakistan Kidney and Liver Institute, Lahore, Punjab, Pakistan

**Keywords:** antibodies, COVID-19, maternal immunization, severe acute respiratory syndrome coronavirus 2 (SARS-CoV-2)

## Abstract

**Objectives:**

As coronavirus disease 2019 (COVID-19) continuous to spread, the transfer of maternal anti severe acute respiratory syndrome coronavirus 2 (SARS-CoV-2) antibodies via lactation is an important source of immunity in newborns that requires more comprehensive studies to improve vaccine options in these candidates. The aim of this study was to evaluate SARS-CoV-2 spike protein antibodies against COVID-19 in breast milk and serum of lactating mothers post vaccination and to establish a correlation between both.

**Methods:**

Hundred and eighty lactating mothers were included in this cross sectional cohort study conducted at Rehman Medical Institute, Peshawar. We described the immunogenicity 21 days after the booster dose of vaccine in 21 patients. Breast Milk and serum specimens were collected and investigated for SARS-CoV-2 spike protein antibodies by consuming electro-chemiluminescence immunoassay (Elecsys Anti-SARS-CoV-2 S Roche, Switzerland).

**Results:**

One-hundred percent of patients revealed robust positive findings to SARS-CoV-2 spike proteins antibodies in breast milk and 85 percent in serum, i.e., >0.8 IU/mL. Our study shows that lactating mothers can mount robust immune reactions against SARS-CoV-2 post vaccination.

**Conclusions:**

All participants had significantly higher antibody titers against SARS-CoV-2 after vaccination. Participants had antibody titers one scale higher post vaccination than pre vaccination. A significant correlation was found between SARS-CoV-2 antibodies in milk and serum. Constant monitoring of antibodies titers is estimated to attain significant humoral immunity against SARS-CoV-2 infection.

## Introduction

Breast-feeding mothers are considered as one of the high-risk groups for coronavirus disease 2019 (COVID-19) vaccination due to the fact of viral transmission to newborns and the possible vaccine side effects in this group. Therefore, no data were available on the vaccination of these participants earlier. Inactivated COVID-19 virus is being used by Sino pharm and Sinovac to elicit an immunological response. Both of them utilize aluminum hydroxide as an adjuvant. Sino pharm and Sinovac were proven to be 73% (40,000 participants) and 83.5% (10,000 individuals) effective in phase III trials, respectively [1], [2], [3]. Sino pharm and Sinovac vaccines were approved for maternal immunization by the World Health Organization (WHO) on the 7th of May 2021 and 1st June 2021, respectively [4, 5]. The Chinese government also highlighted the significance of using these vaccines in this particular group [3, 6, 7]. Research has revealed that anti-severe acute respiratory syndrome coronavirus 2 (SARS-CoV-2) antibodies can be found in the milk of infected mothers [8] but only a few evidence are available of the presence of SARS-CoV-2 spike antibodies presence in immunized lactating mothers [1, 9], [10], [11], [12], [13].

We aimed to evaluate SARS-CoV-2 spike antibody levels in breast milk and serum after maternal immunization and to establish a correlation between the both.

## Materials and methods

After ethical approval from the Institutional Ethical Approval Board, a cross-sectional study was conducted on 180 participants to assess SARS-CoV-2 spike antibody response in post-vaccinated lactating mothers in the Pakistani population from June 1st, 2021 to August 31st, 2021. Among these 21 participants completed the study. All participants received two doses of Sino pharm or Sinovac vaccine 21 days apart. Paired breast milk and serum samples were collected at pre vaccination (time point 1) and 21 days after the second vaccine dose (booster) (time point 2), respectively. A written and informed consent was taken from all subjects prior to sample collection. Vaccination status was validated by a vaccination certificate. Those with the previous history of COVID-19 infection were excluded.

### Collection of breast milk and serum samples

Standard protocols were used to collect breast milk and serum specimens and the presence of SARS-CoV-2 antibodies were detected by using an electro-chemiluminescence immunoassay (ECLIA) (Switzerland) based on double-antigen sandwich assay principle was (Elecsys Anti-SARS-CoV-2 S; Roche Switzerland) kit, for quantitatively determining antibody levels to the SARS-CoV-2 spike protein. Samples were analyzed on Cobas e801 analyzer.

Breast milk samples were collected by participants using breast pumps at residence and then submitted to a lab where they were preserved, processed, and centrifugated. The fat layer was removed and the supernatant was collected and analyzed. Blood samples were collected using acid citrate dextrose, sodium citrate, potassium EDTA, tri potassium EDTA, or lithium heparin tubes. The samples were incubated with biotinylated and ruthenylated RBD antigen, following which streptavidin-coated microparticles were added, and relocated to the measuring cell, where microparticles were magnetically caught onto the exterior of the electrode. The manufacturers recommended serum antibodies cutoff level of 0.8 U/mL, above which is considered positive and vice versa. The diagnostic measuring interval of 0.4–250 U/mL was found to be linearity. Since ECLIA is not yet approved for breast milk samples, therefore, no cutoff point was established. Breast milk is a more heterogeneous sample compared to serum, therefore, mean antibody concentration in the breast milk of unvaccinated (control group) was subtracted from each result to avoid analytical interference.

Descriptive statistics were carried out for all demographics. Correlation analysis was performed between antibody concentrations of serum and breast milk with vaccination status using Pearson correlation.

## Results

The mean maternal age was 31 years (range 24–38) and the mean age for infants was 17 months (range 10–24 months) (Table 1). The test findings revealed positive SARS-CoV-2 spike antibody titers among all of the enrolled patients. Antibody levels were reported using units of U/mL. Hundred percent of patients had positive antibody titers in breast milk post vaccination 128 U/mL. Eighty-five percent of patients had positive antibody titers in serum >0.8 U/mL 21 days after booster dose (Table 2). Participants had significant immunogenic responses post vaccination compared to pre vaccination in breast milk samples; this difference (128 vs. 0.2) was statistically significant (p=0.001). The correlation between antibody concentration post vaccination was also statistically significant in serum samples compared to pre vaccination (p-value 0.001) (Table 3). IgG concentration in serum and breast milk samples are positively correlated (p-value 0.001) (Table 4).

**Table 1:** Maternal and Infants demographic characteristics.

Variables	Number, %
Participants	21
**Maternal average age, years**	31
Range, years	24–38
**Infants average age, months**	17
Range, years	10–24

**Table 2:** Antibody concentrations in serum and breast milk of lactating mothers’ pre and post vaccination.

IgG concentration, U/mL	Serum	Pre vaccination (time point 1)	0.1
IgG concentration, U/mL	Serum	Pre vaccination (time point 2)	54
IgG concentration, U/mL	Breast milk	Pre vaccination (time point 1)	0.2
IgG concentration, U/mL	Breast milk	Pre vaccination (time point 2)	128

**Table 3:** Correlation of maternal vaccination with breast milk and serum antibody titers.

Variables	Sample	rs	p-Value
Pre vaccination	Breast milk	0.8	0.236
Post vaccination	Breast milk	0.141	0.001
Pre vaccination	Serum	−0.11	0.64
Post vaccination	Serum	0.48	0.001

**Table 4:** Correlation between IgG concentration in serum and breast milk.

Variables	rs	p-Value
IgG concentration serum and breast milk	0.84	0.001

## Discussion

The immune responses to SARS-CoV-2 spike antibodies in lactating mothers must be determined in order to comprehend the potential protective implications of COVID-19 maternal vaccination. We initiated and established serological screening on 21 participants to determine IgG quantitative antibodies against Spike protein in breast milk and serum in a Pakistani population. At the time of study two SARS-CoV-2 vaccines were given approval for use in breastfeeding mothers by WHO [4].

All enrolled participants in a study presented remarkably higher anti-SARS-CoV-2 antibody levels in breast milk, the results are comparable to other studies [14].

We also observed a significant rise in SARS-CoV-2 IgG antibodies after the second dose consistent with a study conducted on 84 lactating women in Israel [15]. This encouraging outcome supports the idea that breastfeeding mothers’ vaccination on their infants may have a protective impact similar to a study conducted on 17 lactating mothers in Moscow [16]. Significant higher levels of antibody concentration are observed both in serum and breast milk of lactating mothers in a study reported on 110 lactating mothers in Spain [17]. The SARS-CoV-2 IgG antibodies are significantly correlated in milk and serum samples in a study conducted in Poland on 32 Breastfeeding Health Care workers [18].

The present study is important not only for strategically planning vaccination policy at the organization and national level but also reveal the necessity for accurately determining SARS-CoV-2 spike antibody levels among lactating mothers post vaccination globally. Small sample size was used in the current study.

## Conclusions

The findings of our study imply the following conclusions:

- A maximum number of lactating mothers develops immunity post vaccination with levels rising substantially after the booster dose.
- There was a strong positive correlation between SARS-CoV-2 IgG antibodies in milk and serum samples.
- Although more research is needed but these findings could have a significant impact on vaccination in this particular group.
- These findings would also benefit infants’ immunity against COVID-19.
